# Effectiveness of Pain Neuroscience Education and Therapeutic Exercise in Adults with Musculoskeletal Pain: A Randomized Controlled Experimental Pilot Study

**DOI:** 10.3390/healthcare14213663

**Published:** 2026-10-28

**Authors:** Raquel Reguera-Arzúa, Irimia Mollinedo-Cardalda, Esther Monge-Pereira, Iris Machado de Oliveira, Sebastián Jesús Aboal-Quintás, Orlando Conde-Vázquez

**Affiliations:** 1 Isaúde Research Group, Facultade de Fisioterapia, Universidade de Vigo, 36005 Pontevedra, Spain; raquel.reguera@alumnado.uvigo.gal (R.R.-A.); esther.monge@uvigo.gal (E.M.-P.); irismacoli@uvigo.es (I.M.d.O.); orlando.conde@uvigo.es (O.C.-V.); 2 Studio Pilates Fisioterapia, 36001 Pontevedra, Spain; sjesusaboal@gmail.com; 3 FisioIsaúde Research Group, Galicia Sur Health Research Institute (II Galicia Sur), SERGAS-UVIGO, 36312 Vigo, Spain

**Keywords:** chronic pain, fear-avoidance beliefs, pain education, physical exercise, kinesiophobia

## Abstract

**Highlights:**

**What are the main findings?**

- Adding a structured pain neuroscience education (PNE) programme to therapeutic exercise was not associated, in this pilot sample, with a statistically significant additional reduction in pain intensity or increase in muscle strength beyond exercise alone in adults with chronic musculoskeletal pain.
- PNE combined with exercise produced significantly greater reductions in kinesiophobia and fear-avoidance beliefs than exercise alone, with good adherence and no adverse events.

**What are the implications of the main findings?**

- In real-world rehabilitation settings, the added value of PNE combined with exercise may lie mainly in psychological outcomes rather than in pain intensity or strength gains.
- A larger, adequately powered multicentre randomized trial is warranted to confirm whether PNE provides clinically relevant benefits when added to structured therapeutic exercise.

**Abstract:**

Background/Objectives: Chronic musculoskeletal pain is a common public health problem with substantial functional and socioeconomic impact. Pain neuroscience education (PNE) has been proposed as a complementary strategy to therapeutic exercise; however, the available evidence shows considerable methodological variability and a limited number of studies evaluating its added effect in real-world clinical settings. This study evaluated the effect of incorporating a structured PNE programme into a therapeutic exercise protocol on pain intensity, kinesiophobia, and muscle strength in patients with musculoskeletal pain. Methods: A randomized controlled pilot trial was conducted in patients with chronic musculoskeletal pain. Participants were assigned to an experimental group (exercise + PNE) or a control group (exercise only). Participants were adults with chronic musculoskeletal pain of at least three months’ duration, predominantly women, with pain located mainly in the lumbar region, followed by the neck, shoulder, and knee. The intervention lasted 8 weeks, and pain, kinesiophobia, and muscle strength were assessed before and after the intervention. Outcome comparisons were performed using intra- and intergroup non-parametric statistical analyses, with the significance level set at *p* < 0.05. Results: No significant between-group differences were observed in pain intensity. The group receiving PNE showed greater improvements in kinesiophobia and fear-avoidance beliefs. Both groups significantly improved muscle strength, with no differences between them. Mean attendance at the PNE sessions was 88.3%; 31 of the 38 randomized participants (81.6%) were included in the final analysis, and no adverse events were observed. Conclusions: No statistically significant additional effect of PNE was detected on pain intensity or muscle strength in this pilot sample, although it was associated with greater reductions in kinesiophobia and fear-avoidance beliefs. Larger studies are needed to confirm these findings.

## 1. Introduction

Chronic pain constitutes a major public health problem. Recent studies estimate that approximately 25.9% of the Spanish adult population presents chronic pain, with a higher prevalence among women and middle-aged individuals [1]. Among the different clinical presentations, low back and neck pain are consistently reported as being among the most prevalent and disabling forms of chronic musculoskeletal pain, followed by shoulder- and knee-related pain [1,2]. This condition is associated with a reduction in the quality of life of those who suffer from it, constituting one of the main causes of disability, work absenteeism, and healthcare resource use [2,3]. Moreover, a considerable number of patients show no clear correlation between tissue damage and the pain experience, maintain their pain despite interventions targeting anatomical structures, and develop fear, avoidance, and dysfunctional beliefs [3].

However, clinical practice continues to be dominated by a structural interpretation of pain, in which the intensity of symptoms is directly linked to the magnitude of observable tissue damage [3]. Although this approach can be useful in acute processes, it presents limitations when applied to persistent pain conditions, in which the correlation between structural findings and pain intensity tends to be weak or non-existent [3,4]. This fact suggests the involvement of mechanisms related to central pain processing [4]. For this reason, chronic musculoskeletal pain should not be addressed solely from a tissue-based perspective, but rather requires strategies that modulate physical and social factors [5].

In recent decades, the understanding of pain has evolved from biomedical models focused exclusively on tissue damage toward biopsychosocial models that recognize pain as a complex, multifactorial experience [6]. These models consider the influence of cognitive, emotional, and behavioral factors on pain perception and maintenance, highlighting variables such as beliefs about pain, fear of movement, and avoidance behaviors [3,6]. At the neurophysiological level, these changes are related to a plastic reorganization of the central nervous system, which may affect not only pain processing but also motor control and muscle strength [7,8,9]. In this context, the perception of threat associated with pain has been identified as a key element that can condition patient participation in physical activities and therapeutic interventions [6], which has driven the development of treatment strategies aimed at modifying pain interpretation, among which pain neuroscience education (PNE) stands out [10]. This intervention is based on the structured delivery of knowledge about pain mechanisms, including aspects related to its physiology, the basic mechanisms involved in its perception, and the basic principles for its management in the clinical setting [6]. Through this educational process, PNE aims to promote changes in pain-related beliefs and facilitate patient participation in active treatment programs aimed at functional recovery [6]. Recent research notes that the educational component achieves its full value when combined with therapeutic exercise, since while PNE acts on beliefs and the cognitive interpretation of pain, exercise offers a pathway for progressive exposure to movement [11]. In fact, the available evidence shows that PNE applied in isolation achieves limited and poorly sustained effects over time [12], compared with the greater long-term improvements in pain observed when it is combined with therapeutic exercise [13].

Despite growing interest in PNE as part of the management of persistent musculoskeletal pain, the available evidence presents important limitations that make it difficult to establish firm conclusions about its clinical application. Recent systematic reviews have noted considerable heterogeneity in the content, duration, and format of educational interventions, as well as in their integration within therapeutic programs, which limits comparison between studies and generalization of results [6,12,14]. Moreover, many studies have analyzed PNE as an isolated intervention or as part of poorly standardized multimodal programs, and the number of studies specifically evaluating its added effect when combined with structured therapeutic exercise programs applied in real clinical settings remains limited [15].

The main objective of this study was to evaluate the effect of incorporating a structured PNE program into a therapeutic exercise protocol, compared with therapeutic exercise alone, on pain intensity in patients with chronic musculoskeletal pain. As secondary objectives, the study aimed to estimate the effect of this intervention on kinesiophobia, fear-avoidance beliefs, and muscle strength, as well as to assess the feasibility (recruitment, adherence to exercise and PNE sessions, dropout rate, and safety) of the study protocol with a view to informing a future larger-scale trial.

## 2. Materials and Methods

### 2.1. Study Design

This is a randomized pilot clinical trial with a convenience sample. It was conducted following the guidelines of the Consolidated Standards of Reporting Trials (CONSORT 2025 Statement) [16].

Following the PICO (Population, Intervention, Comparison, Outcome) model, the target population (P) consisted of adult patients with chronic musculoskeletal pain, the intervention (I) was the addition of PNE to a therapeutic exercise program, the comparator (C) was therapeutic exercise alone, and the outcome variables (O) were pain intensity, kinesiophobia, and muscle strength.

### 2.2. Participants and Recruitment

All individuals who were part of the therapeutic exercise working groups in a functional rehabilitation center were invited to participate, forming a sample selected through convenience sampling. During the recruitment phase, participants were provided with detailed information about the research project, including the assessment measures, type of intervention, duration, selection criteria, and study objectives, and were invited to participate.

Participants who met the inclusion criteria defined in the corresponding section were selected. Prior to the start of the study, informed consent was obtained, in which the nature of their participation, as well as the benefits and risks of the intervention, were explained. Personal, health, and demographic data were then collected through an interview in order to verify that participants met the selection criteria.

After recruitment, participants were randomly assigned to the intervention or control group through a simple randomization sequence with a 1:1 ratio using the randomizer.org application. Allocation was performed by a researcher once the baseline assessment had been completed. The random allocation sequence was generated using the randomizer.org web-based application by a member of the research team who was not involved in outcome assessment. The same researcher was responsible for enrolling participants and for performing the allocation, which was carried out only after the baseline assessment had been completed for each participant, thereby concealing group assignment from participants and outcome assessors until that point. Randomization was simple (1:1), without stratification or blocking by sex, age, or any other baseline variable. Given the relatively small sample size, this non-stratified procedure may have contributed to the sex imbalance observed between groups after randomization (see Section 4.1).

### 2.3. Eligibility Criteria

Inclusion criteria: Being over 18 years of age, presenting chronic musculoskeletal pain (>3 months) regardless of its location, and signing the informed consent form to participate in the study. Both men and women were eligible for participation, with no upper age limit; the age of the participants finally included in the analysis ranged approximately between 41 and 59 years, based on the interquartile ranges reported by group in Table 1. Eligibility was confirmed for each participant through the structured baseline interview described in Section 2.2, which systematically collected sociodemographic and clinical information to verify compliance with the inclusion criteria and the absence of any exclusion criterion before enrolment.

Exclusion criteria: Presenting a psychiatric or psychological condition that hinders the execution or understanding of the intervention, both of the exercise program and the educational talks; presenting neurological diseases of a degenerative nature or affecting the central nervous system; having recently undergone surgery; or being pregnant.

### 2.4. Assessment Tools and Study Variables

Assessments were carried out at the Studio Pilates center (Pontevedra, Spain) by three physiotherapists external to the research team who were blinded to participant allocation; assessors were not informed of group assignment at either assessment time point and had no involvement in the delivery of the exercise or PNE sessions, which minimized the risk of unblinding. However, blinding effectiveness (e.g., by asking assessors to guess group allocation) was not formally tested, which is acknowledged as a limitation. Variables were recorded at two time points: before the start of the intervention and after it (week 9).

- Sociodemographic and clinical variables: age, sex, weight, height, and pain location were collected for each participant.
- Outcomes of interest: three scales validated in Spanish were used—the Numeric Pain Rating Scale (NPRS) [17] to assess the maximum pain experienced in the last 7 days; the Tampa Scale for Kinesiophobia (TSK-11) [18] and the Fear-Avoidance Beliefs Questionnaire (FABQ) [19] to assess kinesiophobia and cognitive aspects linked to pain.
- Muscle strength: four representative movement patterns were assessed—a knee-dominant exercise (squat), a hip-dominant exercise (deadlift), an upper-body push exercise (bench press), and an upper-body pull exercise (bench-supported row). Dynamic muscle strength was assessed using a linear velocity transducer (Vitruve, Speed4Lifts S.L., Madrid, Spain), which made it possible to quantify the movement velocity of the load during the execution of exercises with an Olympic barbell. A load equivalent to 70% of the one-repetition maximum (1RM) was used for all exercises [20,21,22], except for the deadlift, for which a load of 80% of 1RM was used [23], since the available literature on reference velocity values for this exercise is mainly described for this intensity. Each participant performed three attempts per exercise, with a five-minute recovery period between them, ensuring a safe and reproducible assessment while minimizing fatigue. For each participant and exercise, 1RM was not measured directly through a single maximal-effort repetition; instead, it was estimated indirectly from the individual load-velocity relationship recorded with the linear velocity transducer, following the procedures described in the load-velocity profiling literature [20,21,22]. The kilogram values reported in the Results therefore correspond to the absolute external load (barbell load) lifted at the prescribed percentage of each participant’s estimated 1RM, rather than to a directly measured maximal load. Of the three attempts performed per exercise, the repetition showing the highest mean propulsive velocity for the corresponding load was retained for analysis, in accordance with standard velocity-based training procedures.

### 2.5. Intervention

Once the assessments had been carried out and participants had been randomly assigned to the experimental and control groups, the intervention phase began, lasting 8 weeks. Both groups followed the same physical exercise program with the same dosage (see Supplementary Material S1). However, the experimental group also received a PNE intervention (see Supplementary Material S2), while the control group carried out only the therapeutic exercise program.

Participation in this study involved minimal risks associated with therapeutic exercise, such as transient muscle soreness or fatigue, and, in the case of PNE, a possible initial increase in attention to pain when new concepts were introduced [24,25]. To minimize these risks, all exercise sessions were supervised by qualified physiotherapists, who adjusted the intensity and progression of the exercise to the individual characteristics of each participant. The protocol allowed for the possibility of modifying or discontinuing the activity in the event of discomfort or any clinical incident, as well as referral to the participant’s usual healthcare professional in the event of significant worsening of pain or functional status. Discontinuing participation would not have entailed any detriment to participants’ usual care.

The experimental group received four PNE sessions, at a frequency of one session every two weeks. This dose (four sessions, one every two weeks, over the 8-week intervention period) was chosen a priori based on feasibility considerations for delivery in a routine clinical setting and on prior PNE protocols using a similarly limited number of sessions; a fortnightly interval was selected to allow participants time to reflect on and apply the concepts covered before the next session, while minimizing additional burden on participants who were concurrently attending exercise sessions. We acknowledge, however, that the optimal dose, session duration, and delivery format of PNE have not been firmly established, and that this relatively low-intensity format may have limited the magnitude of the effects observed, particularly on pain (see Section 4.2 and dose–response evidence [26]). PNE was delivered by one of the researchers, who was unaware of the results of the groups’ baseline assessments (blinded to assessment). The educational intervention was based on PNE principles, aimed at facilitating the reconceptualization of pain through an accessible explanation of the neurophysiological mechanisms involved. The sessions were delivered as theoretical classes in an online format, through videos uploaded to the YouTube platform, in which the currently known neurophysiological mechanisms of pain were explained using simple language. During the sessions, examples were provided and, with the support of graphic material and in an accessible way, the functioning of the excitatory and inhibitory pain mechanisms was explained, from their reception in the periphery to their modulation by the central nervous system. At the end of each session, a brief questionnaire was administered to verify attendance and understanding of the content covered. The videos were pre-recorded and were intended to be viewed independently by each participant at home, at their own pace; approximate session duration ranged between 10 and 15 min. Completion of each session was verified through the brief questionnaire administered immediately afterward, which participants completed and returned to the research team; this questionnaire served both to confirm that the video had been viewed and to check basic understanding of the key concepts presented, and was used as the basis for calculating adherence to the educational component.

Likewise, both groups received 50 min therapeutic exercise classes, with a usual frequency at the center of 1 to 3 sessions per week, aimed at improving strength, muscle coordination, aerobic capacity, and flexibility. The interventions were delivered in group format, with a maximum of four participants per session. These sessions took place at the Studio Pilates center (Pontevedra, Spain) and were delivered by the physiotherapists working there. The intervention consisted of a structured therapeutic exercise program, focused mainly on strength training. This was carried out using free weights, including dumbbells, weightlifting bars, and other similar equipment. In addition, the program included an aerobic exercise component. The frequency, intensity, duration, and progression of training were individualized according to the characteristics and abilities of each participant. The weekly frequency (1 to 3 sessions) was determined individually by the treating physiotherapist according to each participant’s usual availability and clinical status, following the center’s standard practice, and was applied identically to both study groups; as reported in Section 3.7, the number of exercise sessions completed did not differ significantly between groups, supporting a comparable overall exercise exposure despite the individualized weekly frequency. Adherence to the exercise program is reported in Section 3.7 relative to the number of sessions completed during the 8-week intervention period.

### 2.6. Ethical and Legal Aspects

This project was carried out following the ethical principles for medical research involving human subjects established in the Declaration of Helsinki, also ensuring strict compliance with the confidentiality of personal data and results obtained during the study, in accordance with current national regulations.

Participation was entirely voluntary and had no negative impact on the usual care received at the center or on the physiotherapy services provided to those individuals who decided not to participate or to withdraw from the study at any time.

The project was approved by the Research Ethics Committee of the Faculty of Physiotherapy of the University of Vigo under code number 205-2025-04 (28 November 2025). The study was registered at ClinicalTrials.gov (Identifier: NCT07788781). Participant recruitment began on 16 February 2026; the intervention started on 9 March 2026; it was completed on 10 May 2026, and the study was finalized on 5 June 2026. The trial was registered retrospectively on ClinicalTrials.gov on 19 August 2026, after the completion of data collection. This delay resulted from an unintentional oversight in completing the public registration before participant enrolment, for which the authors apologize; the study protocol, including the randomized design, intervention and comparator groups, objectives, and main outcome measures, had nonetheless been finalized and approved by the Research Ethics Committee before recruitment began. The outcomes presented in this manuscript correspond to those specified in the registered protocol.

### 2.7. Data Analysis

A descriptive analysis of the sample and the main study variables was carried out, stratified by allocation group. Quantitative variables were expressed as median and interquartile range (P25–P75), and qualitative variables as absolute and relative frequencies (*n*, %).

The normality of the variables was assessed using Q-Q plots and the Shapiro–Wilk test. Since several variables did not follow a normal distribution, non-parametric tests were used throughout the analysis.

Baseline comparability between groups was analyzed using the Mann–Whitney U test for quantitative variables and Pearson’s chi-square test for qualitative variables, with the *p*-value for the latter reported using Fisher’s exact test. To limit the impact of baseline values within each group, change scores (pre-post difference) were analyzed.

Within-group analysis was performed using the Wilcoxon signed-rank test. Effect size was calculated using Rosenthal’s r coefficient (r = |Z|/√N), with *N* considered as the number of observations. Effects were interpreted as r < 0.3, small; 0.3 ≤ r < 0.5, moderate; and r ≥ 0.5, large. No formal correction for multiple comparisons was applied across the multiple outcomes, subscales, and within- and between-group tests performed in this exploratory pilot study; this increases the risk of Type I error for the secondary and exploratory analyses, and is acknowledged as a limitation in Section 4.7.

**Figure 1:**
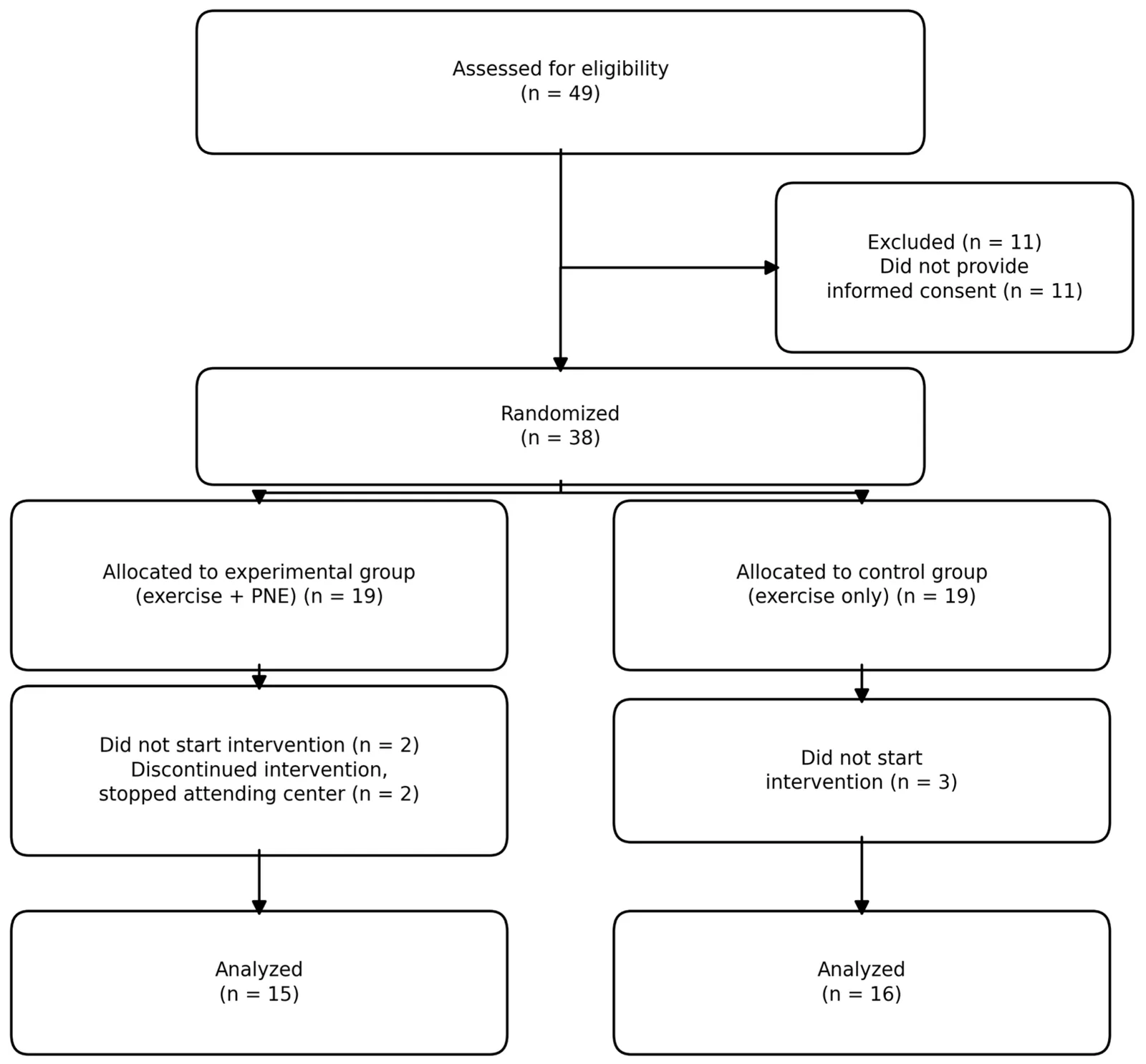
CONSORT (Consolidated Standards of Reporting Trials) flow diagram of participants through the study.

The analysis was performed as a complete-case analysis, including only participants who completed the baseline and post-intervention assessments and who had data available for the study variables at both assessment time points. To minimize missing data, periodic reminders were carried out, and the reason for dropout was recorded whenever possible. Losses to follow-up were recorded and described in the CONSORT flow diagram (Figure 1) [16,27]. An intention-to-treat analysis was not performed because no post-intervention data were available for the seven participants who did not initiate or complete the assigned intervention after randomization (four in the experimental group and three in the control group; Figure 1); no baseline-carried-forward or imputed values were substituted for these missing post-intervention data. We acknowledge that this complete-case approach may introduce attrition bias, particularly given the small sample size, and that the characteristics of the excluded participants were not formally compared with those of participants included in the final analysis; this is discussed further as a limitation in Section 4.7.

As this was a pilot study, no a priori sample size calculation was performed. The sample size was determined based on feasibility considerations and participant availability during the recruitment period. Consistent with the objectives of pilot randomized trials, the study was designed to assess the feasibility of the protocol and to generate preliminary estimates of effect sizes to inform the planning of future definitive trials. Therefore, the study was not powered to detect statistically significant between-group differences, and findings should be interpreted as exploratory. Consequently, non-significant results may reflect insufficient statistical power rather than a true absence of effect. The results obtained are intended to inform sample size calculations and the design of future adequately powered randomized controlled trials. The statistical significance level (α) was set at 0.05 (*p* < 0.05). Statistical analyses and graphs were performed using the IBM SPSS Statistics software package for Windows, version 29 (Armonk, NY, USA: IBM Corporation).

Additionally, to complement the interpretation of statistical significance, a reduction of at least 2 points on the NPRS scale was considered clinically relevant [28,29]. This threshold was used to assess whether the magnitude of the observed change in pain intensity could be considered clinically relevant, regardless of its statistical significance. Participants meeting this threshold were classified as responders and the remaining participants as non-responders. Because responder status is a dichotomous variable, it was reported as the number and percentage of responders in each group and compared between groups using Fisher’s exact test (two-sided), which is appropriate given the small sample size and the expected cell counts below five. Point estimates for between-group differences are reported together with exact *p*-values and effect sizes (Section 3); 95% confidence intervals for the main between-group comparisons were not calculated in the original analysis plan and are noted as a limitation in Section 4.7, to be incorporated in future, adequately powered trials.

## 3. Results

### 3.1. Recruitment and Participants

A total of 49 individuals were assessed for inclusion in the study, of whom 11 were excluded for not providing informed consent. The remaining 38 were randomized in a 1:1 ratio to the experimental group (*n* = 19) or the control group (*n* = 19). In the experimental group, two participants never started the assigned intervention and two others discontinued it after they stopped attending the center, leaving 15 participants for the final analysis. In the control group, three participants did not start the assigned intervention, and 16 participants completed the study and were analyzed. No losses to follow-up or adverse effects were recorded. The final sample analyzed comprised 31 participants (Figure 1).

### 3.2. Baseline Characteristics

Baseline sociodemographic and clinical characteristics are presented in Table 1. The groups were comparable at baseline in age, body mass index (BMI), and pain location (predominantly lumbar in both), with no statistically significant differences. In contrast, significant differences were found in sex (the experimental group consisted entirely of women, versus an even distribution in the control group), weight, and height.

### 3.3. Pain Intensity

In the experimental group (Table 2), pain intensity (measured with the NPRS) decreased significantly by the end of the intervention, while no significant changes were observed in the control group. In the between-group comparison (Table 3), the difference in the magnitude of change did not reach statistical significance, nor did the proportion of responders with a reduction of ≥2 points on the NPRS (experimental group: 7/15, 46.7%; control group: 3/16, 18.8%; Fisher’s exact test, *p* = 0.135).

### 3.4. Kinesiophobia

The experimental group showed a significant reduction in kinesiophobia, with significant decreases in its activity avoidance and harm subscales (Table 2). No significant changes were recorded in the control group for any of these measures. The between-group comparison (Table 3) showed statistically significant differences in favor of the experimental group in the total score and in both subscales, activity avoidance and harm.

### 3.5. Fear-Avoidance Beliefs

In the experimental group, fear-avoidance beliefs related to physical activity (FABQ-PA) decreased significantly, with the total FABQ score at the borderline of significance (*p* = 0.050); the work subscale did not change (Table 2). The control group showed no significant changes in any of the FABQ scores. In the between-group comparison, significant differences were found in favor of the experimental group in the total score, with no significant differences in the physical activity or work subscales (Table 3).

### 3.6. Muscular Strength

Both groups showed a statistically significant increase in load across the four exercises assessed (Table 2). No significant between-group differences were found in the change in any of the strength variables (Table 3).

### 3.7. Adherence to the Intervention

The number of exercise sessions completed (Table 3) was similar in both groups (median of 8 sessions), with no significant differences. In the experimental group, adherence to the educational intervention was high; the median number of PNE talks attended was 4 (3–4), with a mean attendance of 88.3%. Of the 15 participants in the experimental group, nine (60.0%) completed all four sessions, five (33.3%) attended three, and one (6.7%) attended two. Because the weekly exercise frequency was individualized (1 to 3 sessions/week) rather than fixed, a single scheduled-session target was not defined for the cohort as a whole; taking the minimum expected frequency of one session per week over the 8-week intervention as a reference (i.e., a nominal minimum of 8 sessions), the median number of sessions completed (8 in both groups) corresponds to at least full adherence to this minimum reference frequency, without differences between groups. Adherence to the prescribed exercise volume (sets, repetitions, and load progression) within each session was not separately quantified and is acknowledged as a limitation in Section 4.7.

## 4. Discussion

The main objective of this pilot study was to explore the effect on pain intensity in patients with chronic musculoskeletal pain of adding a structured PNE program to a therapeutic exercise protocol, compared with exercise alone. The results showed no statistically significant differences between groups in the primary variable (pain intensity). However, for the secondary variables (kinesiophobia and fear-avoidance beliefs), between-group differences were observed, with greater reductions in some measures in the group that received PNE. These findings should be interpreted cautiously given the pilot nature of the study, the small sample size, and the baseline differences between groups. Regarding muscle strength, both groups improved significantly, with no differences between them. In addition, mean attendance at the PNE sessions was 88.3%; the number of exercise sessions completed was similar in both groups; 31 of the 38 randomized participants (81.6%) were included in the final analysis; and no adverse events were observed. As no feasibility criteria were prospectively established, these data are reported descriptively rather than as evidence that feasibility was formally demonstrated. Throughout this Discussion, we focus primarily on the between-group comparisons, which are the relevant test of a randomized trial’s efficacy; within-group pre-post changes are described only as supporting information and should not, on their own, be taken as evidence of a differential effect between the experimental and control conditions.

### 4.1. Sample Characteristics and Comparability

The final sample consisted of 31 participants with predominantly lumbar chronic musculoskeletal pain, recruited by convenience at a single center. The participants included were mostly women with low back pain, a distribution that partially reflects the current epidemiology of chronic pain in Spain [1], so the characteristics of the sample are broadly consistent with profiles commonly observed in clinical practice. Nonetheless, participants were included regardless of pain location, and the resulting sample combined individuals with low back, neck, shoulder, and knee pain. Although this heterogeneity can be viewed as reflecting the diversity of presentations seen in routine clinical practice (see Section 4.7), it also means that different pain mechanisms, degrees of kinesiophobia, and responses to exercise or to PNE may have coexisted within the same analysis, which could have diluted condition-specific effects and reduced the ability of this pilot study to detect treatment-related differences; this should be considered an additional source of heterogeneity alongside the baseline imbalance in sex described below.

However, the groups were not entirely homogeneous at baseline: although they were comparable in age, BMI, and pain location, they differed significantly in sex (the experimental group was composed entirely of women, whereas in the control group women accounted for only half of the sample). Significant differences were also found in weight and height, which may be attributable to the higher proportion of men in the control group. This imbalance should be taken into account when interpreting the results, particularly those related to strength, since the control group started from higher baseline loads. Analysis of change scores partially mitigates this bias, although it does not eliminate it completely. Because sex may influence not only physical performance but also pain-related and psychological responses, this imbalance may have confounded not only the strength results but also, to some extent, the psychological outcomes (kinesiophobia and fear-avoidance beliefs), and should be borne in mind when interpreting all between-group comparisons in this study, not only those related to strength. Beyond the analysis of change scores, we did not perform additional sensitivity analyses (e.g., adjusting for sex, weight, or height) to further examine the robustness of the between-group findings to this imbalance; this represents an additional limitation, and such adjusted or stratified analyses should be considered in future, larger trials. Accordingly, the present findings should be interpreted with caution, without attributing the observed between-group differences exclusively to the effect of the intervention.

### 4.2. Pain

The absence of significant between-group differences in pain intensity differs from what has been described in the literature. Some meta-analyses [13,15] have found that adding PNE to an exercise program reduces pain in the short term to a greater extent than exercise alone, although with moderate effect sizes and low-quality evidence due to high heterogeneity between studies. In the present study, by contrast, the reduction in pain did not differ between groups, with no statistically significant additional effect of PNE detected in this pilot sample. Moreover, the magnitude of change did not reach the threshold considered clinically relevant (a 2-point or 30% reduction on the NPRS) [28,29], and, although the proportion of responders was numerically higher in the experimental group (46.7% vs. 18.8%), this difference was not statistically significant (Fisher’s exact test, *p* = 0.135).

Several factors could explain this discrepancy: (1) the pilot nature of the study, which was not statistically powered to detect differences in this variable; (2) the low baseline pain intensity (median of 3–4 points on the NPRS), which leaves little room for improvement; (3) the use of a structured, progressive, and supervised exercise program as a comparator, which by itself may have promoted exposure to movement and pain reduction in both groups; (4) the dose and format of the educational intervention, possibly less intensive than that of studies with positive results, in line with the available dose–response analyses [26]; (5) the baseline imbalance in sex, weight, and height between groups (Section 4.1), which may have added variability that further limited the ability of this small sample to detect a between-group difference in pain; and (6) the heterogeneous pain locations included in the sample, since different musculoskeletal pain presentations may respond differently to PNE and to exercise, potentially diluting an average effect that could be more apparent in a more homogeneous population (Section 4.7). In addition, the categorical NPRS-responder analysis (Fisher’s exact test; see Section 2.7) was likely underpowered in this pilot sample, given the small number of participants in each group; this further limits our ability to draw firm conclusions about the absence of a differential responder rate between groups. Taken together, these factors indicate that the absence of a statistically significant between-group difference in pain intensity should not be interpreted as evidence that PNE has no effect on pain; rather, with this sample size, the study cannot rule out a clinically meaningful effect, and the finding is best described as no statistically significant additional effect being detected in this pilot sample.

### 4.3. Effect of PNE on Kinesiophobia

The addition of PNE to the exercise program appears to result in a significantly greater reduction in kinesiophobia than exercise alone, with a moderate effect size on the total TSK-11 score and on both of its subscales, activity avoidance and harm. In contrast, the control group did not show significant changes in any of these scores.

The fact that the added effect was also observed on the harm subscale may be consistent with the content of the intervention, since the central message of PNE—that pain does not necessarily equate to tissue injury—is directly aimed at the beliefs that underlie fear of movement. This pattern of results is consistent with previous findings by Cuenca-Martínez et al. [6] and Medina-Viedma et al. [30], who concluded that PNE reduces kinesiophobia in patients with chronic low back pain. In addition, Kim et al. [31], in their randomized controlled trial in women with chronic low back pain who received PNE added to exercise versus exercise alone, likewise found a significantly greater reduction in kinesiophobia in the group that combined both interventions. Taken together, these findings support the idea that the educational component, by reducing the perceived threat associated with movement, enhances the effect of exercise on fear of movement beyond what exposure to effort achieves on its own [24,25]. Nonetheless, as with the other secondary outcomes, this finding should be interpreted with caution given the baseline imbalance between groups, the small sample size, the absence of correction for multiple comparisons, and the lack of long-term follow-up; the observed reduction in kinesiophobia is therefore best regarded as a preliminary finding warranting confirmation in a larger, adequately powered trial rather than firm evidence of a clinically established added benefit of PNE.

### 4.4. Effect of PNE on Fear-Avoidance Beliefs

For fear-avoidance beliefs, the between-group comparison showed a significant difference in favor of the experimental group in the overall FABQ score. However, when the subscales were analyzed separately, this difference did not reach statistical significance for either physical-activity-related or work-related beliefs. In the within-group comparison, the experimental group significantly reduced physical-activity-related beliefs and showed a decrease at the borderline of significance in the overall score, whereas the control group did not change on any of the scores.

The fact that the between-group difference appears in the overall score but not in each subscale separately suggests that PNE may have a small additional effect on fear-avoidance beliefs, which would be more detectable in the combined measure than in its individual components, where the small sample size limits the ability to detect differences. In addition, both groups followed a structured exercise program which, in itself, constitutes a form of gradual exposure to movement and reduces fear of activity, which may have attenuated the between-group difference on this subscale. In fact, exercise alone has been shown to be capable of reducing fear of movement in people with chronic pain of spinal origin, especially those with chronic low back pain [11]. As for work-related beliefs, most participants started from low or null scores, leaving little room for further reduction, which could explain the absence of change. These results are in line with studies in which adding PNE to exercise improved fear-avoidance beliefs beyond exercise alone [32]. Given the small sample size, the multiple secondary analyses performed, and the borderline *p*-value obtained for the total FABQ score, these findings on fear-avoidance beliefs should be regarded as preliminary and hypothesis-generating rather than conclusive, and require confirmation in an adequately powered trial.

### 4.5. Changes in Muscular Strength

Baseline comparability between groups for these variables was limited; the control group started from significantly higher loads, related to the initial imbalance in sex and, consequently, in weight and height. It should also be clarified that the term ‘muscle strength’ used throughout this manuscript refers to the external load lifted at a given percentage of an indirectly estimated 1RM (a performance-based measure of dynamic lifting capacity under the specific conditions of this protocol), rather than to a directly measured maximal voluntary contraction or an isometric strength value; this distinction, detailed in Section 2.4, should be considered when comparing our results with studies using other strength assessment methods.

Both groups showed a significant increase in the load moved across the four exercises assessed, with no significant differences between them. This result is consistent with the study design, since both groups followed the same strength exercise program, so the improvement in load reflects the adaptation to training common to both conditions. In this regard, a systematic review [33] has shown that posterior-chain strength training produces greater improvements in muscle strength, pain, and disability than general exercise in patients with chronic low back pain.

PNE, in contrast, being a cognitive intervention aimed at beliefs about pain, does not constitute an additional training stimulus capable of increasing strength on its own. In fact, despite the experimental group showing a greater reduction in kinesiophobia, this was not translated into greater strength gains, which supports the idea that strength increases depended on training load rather than on changes in fear of movement. The literature specifically evaluating the added effect of PNE on strength is scarce. One exception is the study by Kim et al. [31], which found a greater improvement in strength in the group that added PNE to exercise, although it was assessed as isometric contraction time of the trunk musculature, which differs from the load applied in this study, limiting direct comparison.

It is notable that the greatest increase occurred in the deadlift and that the squat started from low loads, both exercises that involve a direct load on the spine and that frequently generate apprehension in people with low back pain. In patients with chronic low back pain, the degree of perceived threat regarding a specific task predicts more protective movement behavior [34]. Although the present study did not assess fear specific to performing each exercise, only general kinesiophobia via the TSK-11, the progression of loads in both groups could reflect, in addition to training adaptation, greater confidence in loading the spine as exposure to exercise progressed.

### 4.6. Feasibility of the Study

As a secondary objective, the feasibility of the protocol was assessed given the pilot nature of the study. No feasibility criteria for recruitment, adherence, retention, or dropout had been prospectively defined; therefore, rather than concluding that feasibility was formally demonstrated, we report the observed recruitment, adherence, and retention data below, which may help to inform the design of a larger trial. Adherence to the educational component was high, despite being delivered online. Adherence to the exercise program was similar in both groups, indicating that the addition of PNE did not compromise participation in exercise. Adverse events were monitored throughout the study by the supervising physiotherapists and through participant report during sessions and follow-up contacts; no adverse events were observed during the study period. Given the limited sample size and monitoring methods, we consider it more appropriate to state that no adverse events were observed than to conclude that the intervention has been demonstrated to be safe.

Regarding recruitment, of the 49 individuals assessed, 38 were randomized, and 31 were analyzed, with a dropout rate of close to 18%, mainly attributable to participants who never started the assigned intervention and with no losses during follow-up.

Nevertheless, these results should be interpreted with caution, since participants were recruited at a single physiotherapy center where exercise-based management was already routinely implemented, which makes it difficult to generalize the findings; this setting likely selected participants with relatively favorable attitudes toward exercise and lower baseline fear-avoidance beliefs than might be expected in broader clinical populations less familiar with structured exercise programs. Even so, as this is a pilot study, these findings provide a basis for designing a larger study, with more participants from multiple centers, that can confirm or refute the observed effects.

### 4.7. Limitations

This study has several limitations that should be considered when interpreting the findings. First, the relatively small sample size, inherent to its pilot nature, together with convenience sampling and the absence of a formal a priori sample size calculation, may have reduced the study’s ability to detect statistically significant between-group differences. Therefore, non-significant results should be interpreted with caution, as they may reflect insufficient statistical power rather than a true lack of intervention effect. Accordingly, the findings should be regarded as preliminary and exploratory, serving mainly to provide feasibility data and effect size estimates for the planning of future adequately powered randomized controlled trials.

Second, the groups were not entirely comparable at baseline, with significant differences in sex, weight, and height, which may have influenced both the estimation of the intervention effect and the interpretation of the results, especially for the strength variables. Although the use of change scores partially mitigates this problem by adjusting each outcome to each participant’s baseline status, it does not allow it to be ruled out entirely, so the results relating to muscle strength should be interpreted with caution.

Furthermore, participants were already users of the physiotherapy center (where therapeutic exercise is routinely used), so they had some prior familiarity with exercise and basic prior notions about its role in pain management. This may have resulted in relatively low baseline values for kinesiophobia and fear-avoidance beliefs, reducing the margin for improvement attributable to PNE and limiting the generalizability of the results to people unfamiliar with therapeutic exercise.

The sample also included participants with musculoskeletal pain in different locations, which makes direct comparison difficult with studies focused on a single anatomical region, as is common in the available PNE literature. However, this heterogeneity could be viewed as a strength if it is assumed that, in chronic pain, the pain experience depends more on central mechanisms than on the peripheral location of damage [3,4]; combining different locations could better reflect the applicability of PNE in routine clinical practice, where patients rarely present a single, homogeneous pain pattern. At the same time, as noted in Section 4.1, this heterogeneity should also be regarded as a limitation, since combining several pain presentations in a small sample may have diluted condition-specific effects on kinesiophobia, pain, and strength, and reduced the study’s ability to detect treatment-related differences; both interpretations should be kept in mind when generalizing these findings.

In addition to the above: (1) the online format of the PNE sessions, which may have influenced the degree of adherence; (2) the absence of medium- or long-term follow-up, which makes it impossible to know whether the effects are maintained over time; (3) both the duration of the intervention and the dose and format of the educational component may have been insufficient to achieve greater results, particularly on pain, in line with the available dose–response analyses [26]; (4) the analysis was performed on a complete-case basis, without an intention-to-treat analysis, which may have introduced attrition bias given that seven randomized participants (four in the experimental and three in the control group) did not initiate or complete the intervention and could not be compared with those included in the final analysis; (5) no correction for multiple comparisons was applied across the numerous outcomes, subscales, and statistical tests performed, so some statistically significant findings, particularly among the secondary and exploratory analyses, may represent Type I errors; and (6) 95% confidence intervals were not calculated for the main between-group effects, which would have provided additional information on the magnitude and precision of the observed differences beyond statistical significance alone.

### 4.8. Future Research Directions

The results of this study open up several avenues for research. The main one would be to conduct a larger, multicenter randomized controlled trial with a sample size calculated in advance, to confirm the observed effects. It would also be advisable to include medium- and long-term follow-up to determine whether the effects are maintained over time.

It would also be of interest to study participants with varying degrees of familiarity with exercise, as well as to balance the composition of the groups, in order to improve the representativeness and comparability of the results.

Finally, future research could examine the optimal dose and format of PNE and incorporate other relevant clinical variables, such as disability and quality of life, to provide a more complete picture of the possible added benefit of PNE. It would also be of interest to compare participants receiving PNE with those receiving other cognitive therapies or therapies focused on improving individual resilience.

## 5. Conclusions

In this study, the addition of a PNE program to a therapeutic exercise program was not associated with a statistically significant additional effect on pain intensity or muscle strength in this pilot sample, but it was associated with a greater reduction in kinesiophobia and fear-avoidance beliefs than exercise alone. Taken together, these findings suggest that, in this sample, the added benefit of PNE was concentrated in psychological factors related to pain rather than in pain intensity or muscle strength. It should also be emphasized that although the experimental group showed a statistically significant within-group reduction in pain, this change did not reach the predefined threshold for clinical relevance, underscoring that the observed effects on pain, in either group, were of limited clinical magnitude. Because this is a pilot study, with a small sample size and the limitations noted above, the results should be interpreted with caution and require confirmation in larger trials. The observed adherence (mean attendance of 88.3% at the PNE sessions and a similar number of exercise sessions in both groups), retention (31 of 38 randomized participants analyzed), and the absence of observed adverse events may help to inform the design of a larger, adequately powered trial.

## Figures and Tables

**Table 1:** Baseline sociodemographic and clinical characteristics of participants.

Characteristic	Experimental Group (*n* = 15)	Control Group (*n* = 16)	Statistic	*p*-Value
Age (years)	49 (41–52)	52 (45.25–59.25)	U = 88.000	0.205
Sex–Male	0 (0%)	8 (50%)	χ^2^ = 10.109	0.002 *
Sex–Female	15 (100%)	8 (50%)		
Weight (kg)	64 (57–70)	80 (57.75–89)	U = 68.500	0.041 *
Height (m)	1.62 (1.60–1.65)	1.75 (1.60–1.87)	U = 67.500	0.037 *
BMI (kg/m^2^)	24.51 (20.96–25.91)	25.40 (21.18–27.23)	U = 100.000	0.429
Pain–Knee	1 (6.7%)	0 (0%)	χ^2^ = 1.694	0.757
Pain–Low Back	10 (66.7%)	13 (81.3%)		
Pain–Shoulder	2 (13.3%)	1 (6.3%)		
Pain–Neck	2 (13.3%)	2 (12.5%)		

Note. Quantitative variables expressed as median (P25–P75); qualitative variables as *n* (%). Mann–Whitney U test (U) for quantitative variables; Pearson’s chi-square test (χ^2^) for qualitative variables, with the *p*-value reported using Fisher’s exact test. Body mass index (BMI). A *p*-value < 0.05 was considered significant, indicated with *.

**Table 2:** Within-group statistics.

Variable	Time Point	Experimental (*n* = 15)	*p*-Value	r	Control (*n* = 16)	*p*-Value	r
NPRS	Pre	4 (2–6)	0.022 *	0.42	3 (2–6)	0.729	0.06
	Post	3 (2–4)			3 (2.25–5)		
TSK-11 Total	Pre	20 (18–23)	0.001 *	0.58	19.50 (16.25–26.75)	0.925	0.02
	Post	15 (14–17)			21 (16.25–26.25)		
TSK-11 Activity Avoidance	Pre	12 (8–13)	0.004 *	0.53	11.50 (9.25–16)	0.858	0.03
	Post	9 (8–10)			12.50 (9.25–15)		
TSK-11 Harm	Pre	9 (7–10)	0.002 *	0.55	8 (6–10.75)	0.858	0.03
	Post	7 (6–8)			7.50 (6–10.75)		
FABQ Total	Pre	19 (9–29)	0.050	0.36	8.50 (5–23.75)	0.484	0.12
	Post	12 (8–24)			12 (6.25–29)		
FABQ–Physical Activity	Pre	5 (2–12)	0.026 *	0.41	5 (0.50–9.50)	0.906	0.02
	Post	4 (2–8)			4.50 (0.50–9.50)		
FABQ–Work	Pre	5 (2–18)	0.715	0.07	3.50 (0–15.25)	0.735	0.06
	Post	5 (1–14)			5.50 (0–19.75)		
Deadlift (kg)	Pre	50 (35–55)	0.001 *	0.63	60 (51.25–73.75)	0.001 *	0.61
	Post	60 (55–73)			70 (56.25–93.75)		
Row (kg)	Pre	19.50 (19.50–24.50)	0.001 *	0.61	30 (19.63–39.88)	0.001 *	0.61
	Post	30 (25–35)			33.25 (26.25–53.75)		
Bench Press (kg)	Pre	14.50 (14.50–25)	0.001 *	0.60	25 (15.75–30)	0.002 *	0.55
	Post	20 (20–25)			25 (22.75–42.50)		
Squat (kg)	Pre	20 (19.50–20)	0.001 *	0.58	22.50 (16.13–43.75)	0.001 *	0.59
	Post	25 (20–30)			25 (21.25–52.50)		

Note. Data expressed as median (P25–P75). Within-group comparison (Pre–Post) using the Wilcoxon signed-rank test *p*-value. Effect size calculated using Rosenthal’s r (r = |Z|/√N); interpretation: r < 0.3, small; 0.3 ≤ r < 0.5, moderate; r ≥ 0.5, large. Numeric Pain Rating Scale (NPRS): scored 0–10, higher scores indicate greater pain intensity. Tampa Scale for Kinesiophobia (TSK-11 total): range 11–44, higher scores indicate greater kinesiophobia. Fear-Avoidance Beliefs Questionnaire (FABQ total): each item is scored 0–6; higher scores indicate greater fear-avoidance beliefs. Physical activity subscale (FABQ-PA) includes items 2, 3, 4, and 5; range 0–24. Work subscale (FABQ-Work) includes items 6, 7, 9, 10, 11, 12, and 15; range 0–42. Kilograms (kg). A *p*-value < 0.05 was considered significant, indicated with *.

**Table 3:** Between-group statistics.

Variable	Experimental (*n* = 15)	Control (*n* = 16)	U	Z	*p*-Value	r
Exercise sessions (days)	8 (8–16)	8 (7.25–8)	92.500	−1.173	0.241	0.21
NPRS responders, *n* (%) (≥2-point reduction)	7 (46.7%)	3 (18.8%)	–	–	0.135 ^†^	–
NPRS change	1 (0–2)	−0.50 (−2–1)	75.500	−1.783	0.075	0.32
TSK-11 total change	4 (2–7)	0 (−2–3.75)	57.000	−2.497	0.013 *	0.45
TSK-11 activity avoidance change	3 (0–4)	0 (−1.50–2.50)	69.500	−2.018	0.044 *	0.36
TSK-11 harm change	2 (1–3)	0 (−2–0)	54.500	−2.630	0.009 *	0.47
FABQ total change	2 (0–7)	−0.50 (−8.75–1)	70.000	−1.991	0.047 *	0.36
FABQ physical activity change	1 (0–3)	0 (−1.75–3.25)	83.000	−1.480	0.139	0.27
FABQ work change	0 (0–0)	0 (−5.50–0)	91.500	−1.317	0.188	0.24
Deadlift change (kg)	−10 (−15 to −5)	−10 (−15 to −5)	119.000	−0.041	0.967	0.01
Row change (kg)	−5.50 (−10.50 to −5.50)	−6.75 (−13.88 to −5)	100.000	−0.804	0.422	0.14
Bench press change (kg)	−5.50 (−5.50 to −5)	−5 (−5.50 to −0.63)	92.000	−1.140	0.254	0.20
Squat change (kg)	−5.50 (−10 to −5)	−5 (−10 to −5)	112.000	−0.326	0.745	0.06

Note. Data expressed as median (P25–P75), except for NPRS responders, expressed as *n* (%). Changes were calculated as Pre–Post. For clinical and psychosocial variables, positive values indicate a reduction in score after the intervention; for strength variables, negative values indicate an increase in load. Between-group comparisons were calculated using the Mann–Whitney U test; U, Z, and *p* statistics are presented. Effect size calculated using Rosenthal’s r (r = |Z|/√N); interpretation: r < 0.3, small; 0.3 ≤ r < 0.5, moderate; r ≥ 0.5, large. NPRS responder: participant with a ≥2-point reduction in NPRS. ^†^ Two-sided Fisher’s exact test; U, Z, and r are not applicable to this comparison. Numeric Pain Rating Scale (NPRS). Tampa Scale for Kinesiophobia (TSK-11). Fear-Avoidance Beliefs Questionnaire (FABQ). Kilograms (kg). A *p*-value < 0.05 was considered significant, indicated with *.

## Data Availability

Data are contained within the article. The data are not publicly available due to participants’ privacy.

## Supplementary Materials

The following supporting information can be downloaded at: https://www.mdpi.com/article/10.3390/healthcare14213663/s1, Supplementary Material S1: Design of the Therapeutic Exercise Program; Supplementary Material S2: Structure and Content of the PNE Sessions.

## Abbreviations

The following abbreviations are used in this manuscript:

PNE	Pain Neuroscience Education
NPRS	Numeric Pain Rating Scale
TSK-11	Tampa Scale for Kinesiophobia (11-item version)
FABQ	Fear-Avoidance Beliefs Questionnaire
FABQ-PA	FABQ Physical Activity subscale
BMI	Body Mass Index
1RM	One-Repetition Maximum
CONSORT	Consolidated Standards of Reporting Trials
