# Supplementary material for: Effectiveness of Pain Neuroscience Education and Therapeutic Exercise in Adults with Musculoskeletal Pain: A Randomized Controlled Experimental Pilot Study
Source: Healthcare (Basel). 2026 Oct 28;14(21):3663. doi: 10.3390/healthcare14213663 (PMC13763383; doi:10.3390/healthcare14213663)
Supplement: Supplementary file 1 [file healthcare-14-03663-s001.zip › healthcare-4558287 suppl file S1.pdf]

healthcare MDPI

Supplementary Material S1. Design of the Therapeutic Exercise Program

The therapeutic exercise program was carried out in person and supervised by physiotherapists experienced in therapeutic exercise. Sessions were delivered in group format, with a maximum of four participants per group, in order to ensure adequate technical supervision, individualization of loads, and adaptation of exercises to the clinical characteristics of each participant.

The intervention lasted a total of 8 weeks. Each session lasted approximately 50 minutes, with a frequency of between one and three sessions per week, individually established according to the availability, functional capacity, and tolerance of each participant.

The program was mainly structured around strength training with loads of between 60–80% of 1RM, complemented by mobility exercises, motor control, and aerobic work. The selection of exercises and load progression were individually adapted, taking into account variables such as initial functional level, previous experience with physical exercise, symptoms, and clinical progress during the intervention.

The sessions followed a common general structure made up of four distinct blocks:

1. **Warm-up and activation.** At the beginning of each session, a warm-up period was carried out to prepare the participant for exercise. This block included active mobility exercises, muscle activation, and low-intensity aerobic work through tasks adapted to each participant's abilities.
2. **Main part.** The main block focused on developing strength exercises targeting the main basic movement patterns, including knee-dominant exercises, hip-dominant exercises, and upper-body pushes and pulls. Both multi-joint and accessory exercises were used, employing different implements and external resistances depending on individual needs. The intensity, volume, and complexity of the exercises were progressively adjusted throughout the intervention, prioritizing gradual exposure to movement and physical effort. Progression was carried out according to each participant's clinical and functional tolerance, favoring the performance of exercise within margins of pain considered acceptable and avoiding movement-related avoidance behaviors.
3. **Cardiovascular work and metabolic conditioning.** The program also included blocks of moderate-to-high intensity cardiovascular work and metabolic conditioning, individually adapted to the characteristics and tolerance of each participant. This component was carried out through interval-based tasks and circuits made up of multiple functional exercises, combining global movements and dynamic activities aimed at increasing cardiovascular demand and overall physical activity level. The organization of this block varied throughout the intervention, using different interval training formats such as EMOM (Every Minute on the Minute), AMRAP (As Many Rounds/Repetitions As Possible), and HIIT (High-Intensity Interval Training), among others. The exercises selected were individually adapted according to each participant's functional capacity, symptoms, and previous experience, allowing variables such as volume, intensity, effort duration, and recovery time to be modified. The aim of this block was to promote progressive exposure to physical effort, improve functional and cardiovascular capacity, increase tolerance to movement, and promote a more positive and safe perception of physical activity.

4. **Cool down.** The final part of the session included mobility exercises, breathing work, and lower-intensity activities aimed at facilitating recovery after effort.

Throughout the intervention, active participation of participants was encouraged, promoting an understanding of exercise as a safe and useful therapeutic tool for managing musculoskeletal pain. Individual adaptation of tasks and progressive exposure to movement were also prioritized, favoring increased confidence, perceived capability, and treatment adherence.
