# Supplementary material for: Effectiveness of Pain Neuroscience Education and Therapeutic Exercise in Adults with Musculoskeletal Pain: A Randomized Controlled Experimental Pilot Study
Source: Healthcare (Basel). 2026 Oct 28;14(21):3663. doi: 10.3390/healthcare14213663 (PMC13763383; doi:10.3390/healthcare14213663)
Supplement: Supplementary file 1 [file healthcare-14-03663-s001.zip › healthcare-4558287 suppl file S2.pdf]

healthcare

MDPI

Supplementary Material S2. Structure and Content of the PNE Sessions

The experimental group received four pain neuroscience education (PNE) sessions, delivered at a frequency of one session every two weeks. Sessions were delivered as theoretical classes in an online format, through videos uploaded to the YouTube platform, in which the currently known neurophysiological mechanisms of pain were explained using simple language. During the sessions, examples were provided and, with the support of graphic material and in an accessible way, the functioning of the excitatory and inhibitory pain mechanisms was explained, from their reception in the periphery to their modulation by the central nervous system. At the end of each session, a brief questionnaire was administered to verify attendance and understanding of the content covered.

Session 1. What is pain really?

Main objective: To differentiate the concept of pain from that of tissue damage and reduce the initial fear associated with pain.

Specific objectives:

- Understand what pain is and what its biological function is, differentiating between acute and chronic pain.
- Explain that pain constitutes a protective signal and not a direct indicator of the degree of injury or tissue damage.
- Validate the participant's pain experience, clarifying that the aim of the sessions is not to convey that "the pain is in your head," but rather to promote a better understanding of pain and its modulation mechanisms.
- Introduce the idea that the brain plays a key role in the pain experience.
- Explain some of the factors that can influence pain processing, such as fear, stress, previous experiences, or beliefs associated with pain.

Session 2. Why does pain become chronic?

Main objective: To explain the phenomenon of central sensitization in a simple and understandable way.

Specific objectives:

- Understand why pain can persist even in the absence of an active injury.
- Introduce the concept of central sensitization and explain the mechanisms that contribute to pain chronification.
- Describe the biological, psychological, and social factors that can contribute to the maintenance of pain, including fear-avoidance behaviors and the so-called "pain cycle."
- Introduce therapeutic exercise as an important tool in the management of chronic musculoskeletal pain.

Session 3. Movement as treatment.

Main objective: To reduce fear of movement and physical exercise, promoting therapeutic adherence.

Specific objectives:

- Understand why therapeutic exercise can be beneficial for people with chronic musculoskeletal pain, explaining its effects on pain sensitivity, confidence in movement, and increased sense of control.
- Change the perception of movement as a threat, promoting gradual and safe exposure to it.

- Explain the possibility of experiencing a certain degree of pain during exercise, including the concept of tolerable pain and providing different strategies for monitoring it.
- Introduce the concept of exercise dosage and progression, emphasizing the importance of gradual progression, consistency, and individualization of the intervention.

Session 4. What can we do to better manage our pain?

Main objective: To promote self-efficacy and encourage the patient's autonomy in managing their pain.

Specific objectives:

- Review the different factors that can modulate the pain experience, avoiding blaming approaches, and noting that pain is usually influenced by the interaction of multiple factors.
- Provide practical self-management strategies, including regular physical activity, progressive exposure to movement, and the management of relapses.
- Normalize the occurrence of fluctuations or transient increases in pain during the recovery process, explaining that these do not necessarily represent a therapeutic failure, and offering guidance on how to respond to these situations.
- Promote patient empowerment and their active participation in the therapeutic process.
